# Hemangioblastoma located in the posterior incisural space mimicking a tentorial meningioma: a case report

**DOI:** 10.1186/s13256-016-0962-z

**Published:** 2016-06-23

**Authors:** Alejandra T. Rabadán, Diego A. Hernández, Leonardo Paz

**Affiliations:** Institute of Medical Research A Lanari, University of Buenos Aires, Combatiente de Malvinas 3150. 1427, Buenos Aires, Argentina

**Keywords:** Hemangioblastoma, Infratentorial supracerebellar approach, Pineal region, Posterior incisura, Case report

## Abstract

**Background:**

The most common type of vascularized tumor located in the posterior incisural space in older patients is the falcotentorial meningioma. Solid hemangioblastomas are rarely found in this particular area of the brain. To the best of our knowledge, the case of only one patient harboring a hemangioblastoma not associated with Von Hippel-Lindau disease has been previously reported in the literature in this anatomic region. Regarding age presentation, it is rare for sporadic hemangioblastoma in any part of the brain to occur in older patients; only two cases have previously been reported, and neither were in this anatomical space. A solid hemangioblastoma represents a surgical challenge because of its high vascularization, very similar to an arteriovenous malformation, and it should be removed *en bloc* to prevent significant intraoperative bleeding.

**Case presentation:**

We report here the case of a 63-year-old white male patient with a sporadic hemangioblastoma located in the posterior incisural space mimicking a tentorial meningioma. It was completely removed *en bloc* via an infratentorial supracerebellar approach with an excellent outcome.

**Conclusions:**

A hemangioblastoma should be considered among the differential diagnosis of hypervascularized masses in the posterior incisural space, even in cases of solid tumors, in older patients, or in the absence of Von Hippel-Lindau disease. These tumors located in the posterior incisural space represent a challenge, and the infratentorial supracerebellar approach provides panoramic exposure to allow safe resection.

## Background

In older patients, hypervascularized tumors of the posterior incisural space associated with the tentorium are commonly tentorial meningiomas. Hemangioblastomas (HBL) are rarely found in this location, not even in older patients [1–4]. We report the case of a patient in his seventh decade of life harboring a sporadic HBL. The angiomatous tumor was a purely solid mass located in his posterior incisural space. To the best of our knowledge, this is only the second reported case of HBL not associated with Von Hippel-Lindau (VHL) disease in this anatomic region, and the first mimicking a tentorial meningioma [3].

The potential risk of significant intraoperative bleeding exists in cases in which the diagnosis has not been suspected preoperatively or not recognized intraoperatively. Because the presumptive diagnosis of HBL has a large impact on the surgical technique, the objective of this report is to emphasize that HBL should be considered among the differential diagnosis of hypervascularized tumors in the posterior incisural space even in cases of a solid mass, in older patients, or in the absence of VHL disease.

## Case presentation

**Fig. 1:**
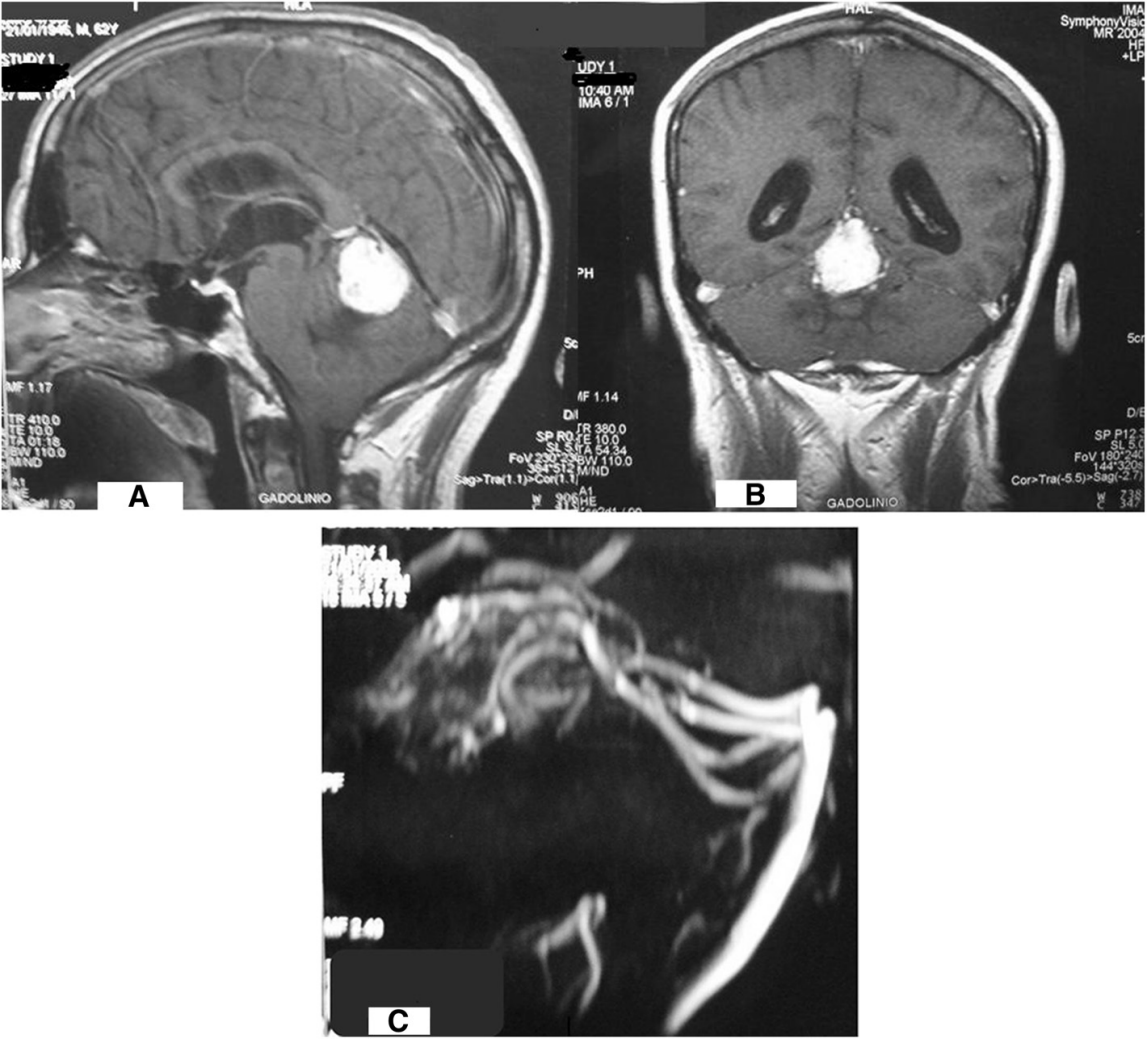
**a** and **b** Gadolinium-enhanced T1-weighted magnetic resonance imaging demonstrated a homogeneously enhancing mass in the posterior incisural space. Coronal and sagittal views. **c** Magnetic resonance angiography showing dilated veins arising from the mass, consistent with a highly vascularized lesion

A 63-year-old white male patient of Polish descent had a 3-month history of headaches, vomiting, and episodes of blurring vision, compatible with intracranial hypertension syndrome. On admission, he had papilledema, nystagmus, and lethargy. A T1-weighted magnetic resonance imaging (MRI) scan showed a low signal intensity lesion in the anterosuperior part of the posterior fossa in contact with the falcotentorial dura junction, displacing inferiorly the cerebellar vermis and compressing the aqueduct of Sylvius, producing obstructive hydrocephalus. The mass enhanced homogeneously with gadolinium-based contrast. Flow voids compatible with vessels were observed around the tumor. (Fig 1a and b). Magnetic resonance angiography (MRA) showed the intense tumoral vascularization (Fig 1c). MRI of the neuroaxis did not reveal any other lesions. Results from whole-body contrast-enhanced computed tomography (CT) scans were normal. His serum levels of alpha-fetoprotein, human chorionic gonadotropin, and carcinoembryonic antigen were normal (Table 1).

**Table 1:** Summary of the case report

1. Patient information	63-year-old white male patient
2. History	3-month history of intracranial hypertension syndrome
3. Relevant physical examination at admission	Papilledema, nystagmus, and patient became lethargic.
4. MRI	T1-weighted MRI showed a hypointense lesion in the anterosuperior part of the posterior fossa in contact with the falcotentorial dura junction, displacing inferiorly the cerebellar vermis and compressing the aqueduct of Sylvius, producing obstructive hydrocephalus. The mass enhanced homogeneously with gadolinium. Flow voids compatible with vessels were observed around the tumor.
5. MRA	Intense tumoral hypervascularization.
6. MRI of the neuroaxis	There were no other lesions.
7. Contrast-enhanced CT scans of the whole body	No relevant findings were found.
8. Laboratory testing	Serum levels of alpha-fetoprotein, human chorionic gonadotropins, and carcinoembryonic antigen were normal.
9. Interventions	
a. First surgical step	An urgent ETV. After the ETV, the patient did well.
b. Second surgical step	Five days later, our patient was operated on. Surgery was performed in a semi-sitting position via an infratentorial supracerebellar approach.
c. Surgical findings	The tumor was a highly vascularized and well-circumscribed mass. A meticulous dissection was performed surrounding the lesion. Several small vessels feeding the tumor were coagulated. The mass reduced dramatically its size, and *en bloc* resection could be performed.
10. Outcome	There were no complications, and our patient had complete recovery of preoperative symptomatology.
11. Pathology	Demonstrated an HBL (Techniques: HE, PAS, Gomori, CD34 immunohistochemistry).
12. Postoperative screening for VHL disease (clinical, laboratory, ultrasound, and images)	Negative according to Melmon and Rosen’s diagnostic criteria for VHL disease [9]. A blood test for the VHL gene, a tumor suppressor gene located at chromosome 3p25-26, was not performed.
13. Long-term follow-up	A 5-year follow-up was uneventful.

*CT* computed tomography, *ETV* endoscopic third ventriculostomy, *HE* hematoxylin and eosin stain, *MRA* magnetic resonance angiography, *MRI* magnetic resonance imaging, *PAS* periodic acid–Schiff stain, *VHL* Von Hippel-Lindau

**Fig. 2:**
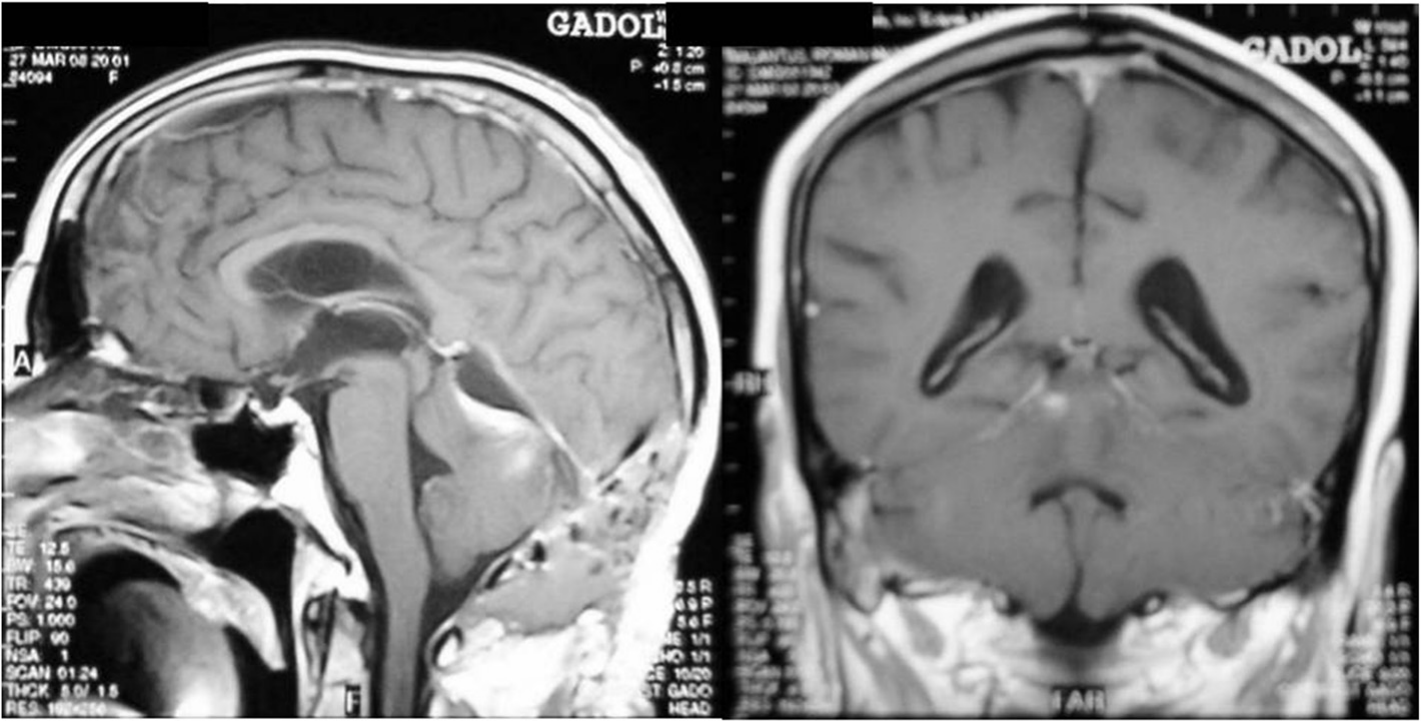
Postoperative gadolinium-enhanced T1-weighted sagittal and coronal magnetic resonance images showing complete mass resection

An urgent endoscopic third ventriculostomy (ETV) was performed to treat the hydrocephalus. After the ETV, our patient did well. Five days later, he was operated on for tumor removal. The surgery was performed in a semi-sitting position via an infratentorial supracerebellar approach. The tumor was a highly vascularized and well-circumscribed mass. The lesion was meticulously dissected from the surrounding tissue. The tumor was found to be separated from the superior aspect of the cerebellar vermis by a thick arachnoidal layer. Several small vessels feeding the tumor and coming from the tentorium were coagulated. The mass dramatically reduced its size, allowing *en bloc* resection to be performed (Fig 2).

**Fig. 3:**
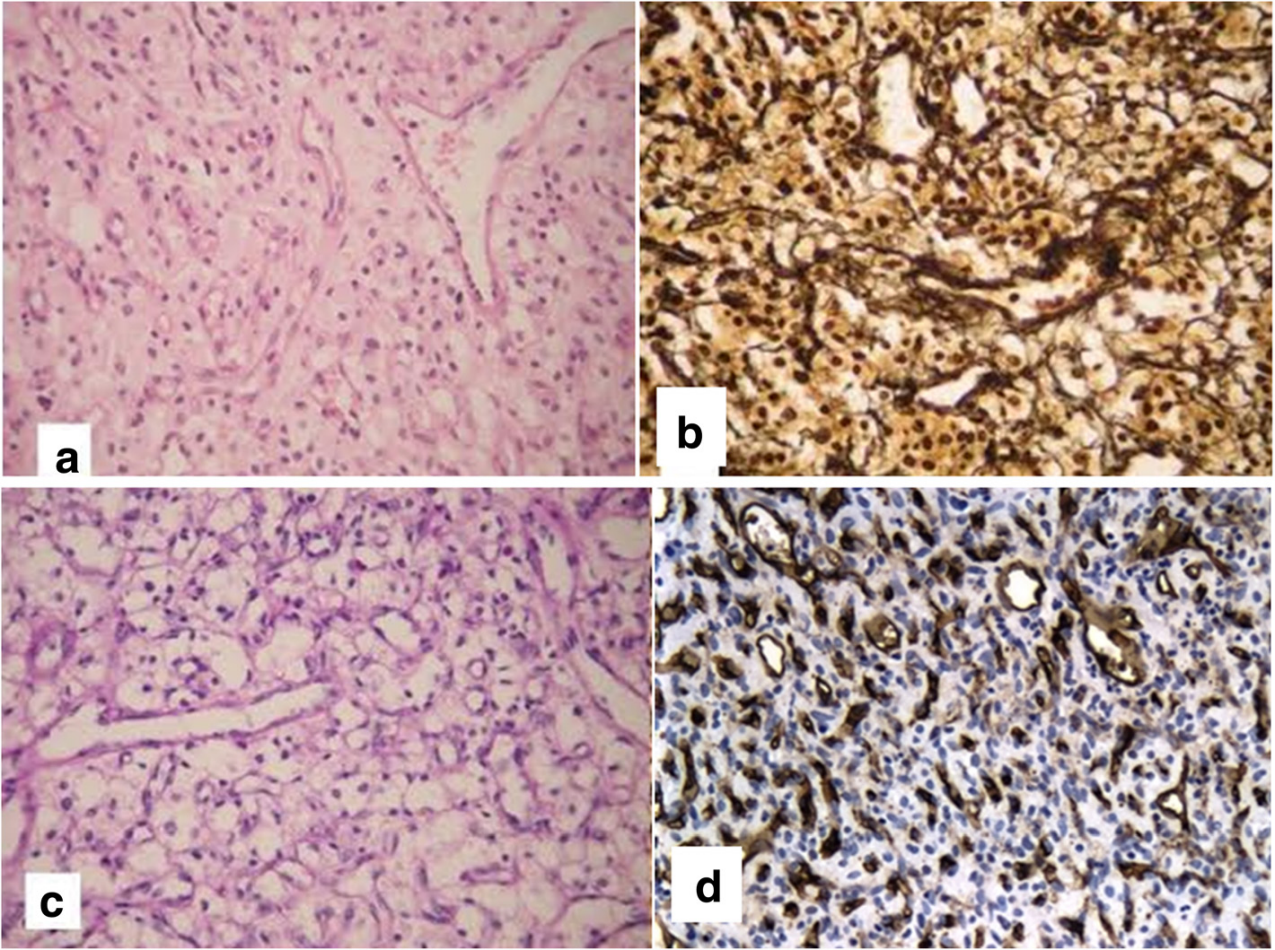
Histopathology. **a** Double component, vascular and cellular (hematoxylin and eosin ×100). **b** Extensive perivascular and intercellular reticulin net (Gomori ×400). **c** Cells with vacuolated and wide cytoplasm in the nidus (periodic acid–Schiff stain ×400). **d** Vessels of different caliber and thin walls (immunohistochemistry with CD 34 stain)

A pathological examination demonstrated a tumor characterized by a double component, vascular and cellular, with a network of small and delicate vessels that separated groups of large polygonal cells, with clear and vacuolated cytoplasm and small, oval hyperchromatic nuclei. A reticulin stain and CD34 immunostain readily delineated the complex capillary networks of the HBL (hematoxylin and eosin, Gomori, periodic acid–Schiff stain, and CD34 immunohistochemistry) (Fig 3).

There were no complications, and our patient had a complete recovery of his preoperative symptomatology. Postoperative screening using clinical, laboratory, ultrasound, and body imaging scans for the detection of VHL disease were negative. A long-term follow-up until 5 years post surgery was uneventful.

## Discussion

HBL is a histologically benign tumor that is associated with VHL disease in 20 % of cases but sporadic in 80 % of cases [1, 5]. Developmentally arrested HBL progenitor cells in the molecular layer of the cerebellum may progress to a tumor in patients with VHL, which is an autosomal dominant cancer predisposition syndrome caused by germline mutations in the VHL gene, a tumor suppressor gene localized at chromosome 3p25-26 that is responsible for tumors in different parts of the body. Patients with VHL syndrome are at risk for the development of new lesions over their lifetime, such as cysts in the pancreas, kidney, broad ligament, and epididymis, as well as clear cell renal cell carcinoma, pheochromocytoma, and/or retinal angiomas. These patients require a lifelong follow-up [6]. Given that a simple peripheral blood test can determine the presence of the VHL gene mutation, we recommend that this study is routinely performed for all patients with an HBL to guide follow-up.

The gene may also be mutated in 4–14 % of cases of sporadic HBL [6]. For sporadic cases of HBL, Melmon and Rosen’s diagnostic criteria for VHL disease continue to be valid in the preoperative work-up [7–10].

HBL usually occurs in young adults, with a mean age at diagnosis of 32 years for VHL-associated HBL and 40 years for sporadic HBL [1, 5]. We found only one paper in the literature reporting (two cases of) sporadic cerebellar HBL in older patients; in neither reported case was the HBL localized to the posterior incisural space as in our patient [2]. Regarding the age presentation, it has been proposed that patients with VHL have a heterozygous recessive tumor suppression gene mutation that requires a single mutation to develop a tumor. In contrast, in sporadic HBL, two spontaneous mutations are required, and this might be the cause of the later presentation in life [11].

HBLs are more frequently found in the cerebellum or in the brain stem; other localizations such as supratentorial, spinal cord, orbit or sellar sites are less common [12]. To the best of our knowledge, only one case has been published reporting a patient harboring an HBL not associated with VHL disease in the posterior incisural space [3]. In our case, the remarkable intraoperative findings were that the arterial feeding came from the tentorium and the visualization of the dural attachment mimicking a tentorial meningioma.

HBL characteristically presents as a cystic mass with a solid mural enhancing nodule; however, presentation as a highly vascular solid mass is not infrequent. Calcifications and edema are rare [13, 14]. Solid HBLs represent a surgical challenge because of their vascularization, similar to an arteriovenous malformation, and this characteristic is more relevant in special areas of the brain such as the posterior incisura [1, 15]. The surgical approach should be wide enough to allow circumferential dissection, coagulation, and division of tumor afferents. Bloodless *en bloc* removal is then possible. Preoperative embolization is not recommended because of poor results [16]. HBL is not radiosensitive, and for this reason stereotactic radiosurgery might only be used to treat small, multiple, or residual HBL [17].

## Conclusions

Although uncommon, HBL is a tumor that should be considered when dealing with vascularized tumors in the posterior incisural space, even in relation to the dura, and at any age. This consideration may help in planning surgical approaches that address the inherent risks of this entity.
